# Variations in Crowding and Ambulance Diversion in Nine Emergency Departments

**DOI:** 10.1111/j.1553-2712.2011.01149.x

**Published:** 2011-09

**Authors:** Daniel A. Handel, Jesse Pines, Dominik Aronsky, Nicholas Genes, Adit A. Ginde, Jeffrey Hackman, Joshua A. Hilton, Ula Hwang, Michael Kamali, Emilie Powell, Medhi Sattarian, Rongwei Fu

**Affiliations:** Center for Policy and Research in Emergency Medicine, Department of Emergency Medicine, Oregon Health and Science University (DAH, RF), Portland, OR; the Department of Emergency Medicine, George Washington University School of Medicine, Department of Health Policy, George Washington School of Public Health (JP), Washington, DC; the Department of Biomedical Informatics & Emergency Medicine, Vanderbilt University (DA), Nashville, TN; the Department of Emergency Medicine, Mount Sinai School of Medicine (NG, UH), New York, NY; the Department of Emergency Medicine, University of Colorado Denver School of Medicine (AAG), Aurora, CO; the Department of Emergency Medicine, University of Missouri–Kansas City, Truman Medical Center (JH), Kansas City, MO; the Department of Emergency Medicine, University of Pennsylvania (JAH), Philadelphia, PA; Geriatric Research, Education and Clinical Center, James J. Peters VAMC (UH), Bronx, NY; the Department of Emergency Medicine, University of Rochester (MK), Rochester, NY; the Department of Emergency Medicine, Northwestern University Feinberg School of Medicine (EP), Chicago, IL; the Department of Emergency Medicine, George Washington University (MS), Washington, DC; and the Department of Public Health and Preventive Medicine, Oregon Health and Science University (RF), Portland, OR.

## Abstract

**Objectives:**

The primary study aim was to examine the variations in crowding when an emergency department (ED) initiates ambulance diversion.

**Methods:**

This retrospective, multicenter study included nine geographically disparate EDs. Daily ED operational variables were collected during a 12-month period (January 2009 to December 2009), including total number of ED visits, mean overall length of stay (LOS), number of ED beds, and hours on ambulance diversion. The primary outcome variable was the ‘‘ED workload rate,’’ a surrogate marker for daily ED crowding. It was calculated as the total number of daily ED visits multiplied by the overall mean LOS (in hours) and divided by the number of ED beds available for acute treatment in a given day. The primary predictor variables were ambulance diversion, as a dichotomous variable of whether or not an ED went on diversion at least once during a 24-hour period, diversion hour quintiles, and sites.

**Results:**

The annual ED census ranged from 43,000 to 101,000 patients. The percentage of days that an ED went on diversion at least once varied from 4.9% to 86.6%. On days with ambulance diversion, the mean ED workload rate varied from 17.1 to 62.1 patient LOS hours per ED bed among sites. The magnitude of variation in ED workload rate was similar on days without ambulance diversion. Differences in ED workload rate varied among sites, ranging from 1.0 to 6.0 patient LOS hours per ED bed. ED workload rate was higher on average on diversion days compared to nondiversion days. The mean difference between diversion and nondiversion was statistically significant for the majority of sites.

**Conclusions:**

There was marked variation in ED workload rates and whether or not ambulance diversion occurred during a 24-hour period. This variability in initiating ambulance diversion suggests different or inconsistently applied decision-making criteria for initiating diversion.

The concept of ambulance diversion was first reported in New York City 20 years ago as a way for ambulances carrying patients with minor injuries to be diverted away from crowded emergency departments (EDs).^1^ The use of diversion in response to ED crowding was reported the following year as a widespread issue in teaching hospitals.^2^ Since that time, diversion has become a common practice, and in many places, the norm rather than the exception as a mitigating method to alleviate the ED crowding burden.^3^ By 2003, the National Hospital Ambulatory Medical Care Survey (NHAMCS) data demonstrated that 45% of EDs initiated ambulance diversion at least once during the previous year.^4^ While diversion may reduce the influx of new ambulance patients into the ED for short periods of time, giving some respite to ED staff to handle active cases, high levels of hospital diversion have been associated with delays in care for critically ill patients with acute myocardial infarction, and, in general populations of patients with chest pain.^5,6^ The relationship between diversion and worse outcomes illustrates that the practice of diversion represents an important public health issue.

In a 2003 commentary, Asplin^7^ asked the question, ‘‘Does ambulance diversion matter?’’ At that time, ambulance diversion was not perceived as a public health threat, was accepted as a standard practice of ED operations, and was seen as a localized problem that EDs and hospitals would need to address. The same year, a study at Syracuse described the first attempt to reduce ambulance diversion.^8^ Many other local and regional efforts across the country followed this example.^9–13^

Until now, systematic approaches to ending diversion have been limited. In 2006, a group of Boston teaching hospitals decided to ban ambulance diversion for 2 weeks. They found no significant effect on ED operations or emergency medical services (EMS) efficiency.^14^ As a result of this finding, the entire state of Massachusetts banned the practice of ambulance diversion on January 1, 2009. Subsequent evaluations from this intervention demonstrated that crowding levels have remained the same,^15^ while EMS arrivals increased slightly.^16^ At the time of this study, a national standard or systematic approach to ending ambulance diversion across the country does not exist.

Ambulance diversion is a major concern across the United States.^17^ Little is known, however, about how individual EDs decide to use diversion, thus limiting the ability to use it as an outcome measure when comparing hospitals. To the best of our knowledge, this is the first multisite study of EDs from across the country, both in community and in academic settings, to examine the variation in whether ambulance diversion occurs within one site and across sites. Unlike most studies in the ED crowding literature that have looked for factors associated with ambulance diversion, i.e., using diversion as the dependent variable, the premise of this study was to compare workload rates among EDs on days with or without diversion to test if differences in workload within and between EDs were associated with diversion.

We determined if the level of ED workload differed on days with and without diversion and evaluated whether or not ambulance diversion reflected ED workload. Our a priori hypothesis was that workload rates would be higher on diversion days when compared to nondiversion days, but that the levels and differences in workload rate would vary between days with and without diversion and between institutions, suggesting a more subjective process of determining when diversion was needed. Thus, unlike most studies in the ED crowding literature that have used diversion as the outcome variable, the goal of our investigation was to evaluate diversion as a predictor of variable ED workload rates.

## METHODS

### Study Design

This retrospective study collected daily ED operational and inpatient hospital variables from nine EDs (eight academic and one community) from geographically disparate regions across the United States. The institutional review board of each participating institution either approved the study protocol or exempted it from review.

### Study Setting and Population

The EDs were from eight different states in four time zones of the United States. All EDs were nonprofit, nongovernmental institutions. Sites saw both pediatric and adult populations with varying percentages. Five of the nine sites had objective diversion criteria. All data were from January 1, 2009, to December 31, 2009. Participating study sites were selected based on their geographic diversity, range of annual censuses, the interest of local investigators, and the fact that ambulance diversion had occurred in their ED during the study period. Study sites needed to be able to provide daily, ED-level data to be included.

### Study Protocol

A standardized template was developed to collect the data and sent to each site. Each site was responsible for compiling its own data and sending them back to the data coordinating center. The data coordinating center reviewed the submitted data to assure consistency in terms of measurement units before being compiled. Daily variables included the total number of ED patients, mean ED overall length of stay (LOS), number of ED beds, and the number of hours an ED was on ambulance diversion each day. The total number of ED patients included both adult and pediatric patients seen each day, and the calculation of mean overall LOS also included both adult and pediatric patients. LOS was defined as the time from first arrival in the ED to when the patient left the ED for his or her final destination. In addition, boarding time was available from five sites and included in secondary analysis. It was defined as the time from when the admission order was placed to when the patient left the ED. All data were compiled in Microsoft Excel (Microsoft Corp., Redmond, WA).

The participating EDs have different capacities and throughput metrics and were not directly comparable using ED level crowding variables. To compare the EDs, we created a daily ‘‘ED workload rate’’ as a standardized marker for ED crowding and used it as the primary outcome variable. The ED workload rate was calculated as the total number of daily ED patients multiplied by the overall mean LOS (in hours) and divided by the number of ED beds available for acute treatment. ED beds were defined as any bed consistently used for treatment, which may have included hallway and fast track beds (if available) at each respective site. The ED workload rate was based on the concept of an occupancy rate, which has been well described in the literature^18^ and has been found to be a valid measure of ED crowding.^19,20^ However, occupancy rate is an hourly variable, and given the limitation of daily data, a different metric was needed. To our knowledge, no other current crowding score exists to look at daily variables, hence necessitating the creation of a new measure. In addition to workload rate, boarding time (in hours) was analyzed as a secondary outcome variable.

The primary independent variable was ambulance diversion used as a dichotomous variable of whether or not an ambulance diversion episode occurred during the 24-hour workload period from midnight to midnight. We also categorized ambulance diversion as a five-level variable (no diversion, between 0 and less than 2 hours, 2 to less than 5 hours, 5 to less than 10 hours, and 10 or more hours) to further assess the association with ED workload rate and the amount of ambulance diversion. These categories were roughly based on a quartile distribution of diversion hours.

### Data Analysis

Institution characteristics were summarized by descriptive statistics. An analysis of variance (ANOVA) model with Bonferroni adjustment for multiple comparisons was applied to determine the differences in workload rate and boarding time, separately. For the primary analysis, which included variables supplied by all nine sites, both main effects and interaction terms between sites and the dichotomous ambulance diversion variable were entered into the model to assess the differences in workload rate among sites on days with and without periods of ED diversion, as well as whether the differences between diverted and nondiverted days differed by sites. A similar model was used for boarding time. A third model was fitted using both the five-level ambulance diversion and sites as main effects only, to examine the association between ED workload rate and the amount of ambulance diversion after adjusting for differences in sites. All models controlled for month, seasonality, and whether or not the day was on a weekend. Model assumptions of normality and homogeneity of variances were checked using Q-Q plot and plot of predicted workload rate versus residuals and were adequately satisfied. Model diagnostic was also performed to check influential or outlying values. All analyses were performed using SAS version 9.1.3 (SAS Institute, Cary, NC)

## RESULTS

The annual census among the nine participating EDs ranged from 43,028 to 101,236 patients. The number of ED beds ranged from 27 to 95. Only one site reported a change in the number of ED beds available during the study period. All sites except one were urban academic settings. The percentage of days that an ED was on diversion at least once during that 24-hour period varied from 4.9% to 86.6% (Table 1).

Table 2 reports the mean ED workload rate by sites and diversion status and the difference in workload rate by diversion status. The crude mean daily ED workload rate varied from 17.1 to 62.1 patient-LOS hours per ED bed on days with ambulance diversion (p < 0.001) and from 14.7 to 59.3 patient-LOS hours per ED bed on days without diversion (p < 0.001) among the different sites.

The mean ED workload rates after adjusting for month and weekday versus weekend were very similar to crude means and varied across sites on both days with ambulance diversion (p < 0.001) and days without ambulance diversion (p < 0001). Overall, differences in workload rates between days with and without ambulance diversion differed across sites (p < 0.001), ranging from 1.0 to 6.0 patient-LOS hours per ED bed. The percentage increase in workload rate between days with and without diversion ranged from 2.2% to 26.1%. Based on the results of multiple comparisons, five of the nine sites had a difference between days with and without ambulance diversion that was statistically significant (Table 2). The five sites that were able to provide boarding times demonstrated increased boarding times on days with diversion. Overall differences in boarding times between days with and without ambulance diversion varied by sites (p < 0.001), and three of the five had statistically significant differences between days with and without diversion (Table 3).

Examining the ANOVA model using the five-level ambulance diversion variable, after adjusting for sites, month, and weekend status, mean workload rates were different among the five levels (p < 0.001). Compared to days without diversion, days with diversion all had significantly higher mean ED workload rate. The mean (±SD) ED workload rate was 1.5 (±0.5) and 2.0 (±0.5) patient-LOS-hours per ED bed higher, respectively, on days with less than 2 hours on diversion and between 2 and 5 hours on diversion, followed by 4.1 and 4.7 patient-LOS-hours per ED bed higher on days with 5 to 10 hours and 10+ hours on diversion, respectively. The results suggest a dose–response relationship with higher mean ED workload rates on days with more hours on diversion.

## DISCUSSION

We found statistically significant differences in the workload rate on days with diversion compared to days without diversion among five of the nine sites.At these five sites, days with ambulance diversion were more crowded than nondiversion days, as indicated by greater ED workload rates. However, the magnitude of the differences was highly variable among the sites. This suggests that there is large variability in what constitutes an ED environment where ambulance diversion is initiated. What is construed to be a situation that requires diversion at one ED may not be interpreted the same way at the same site on a different day (due to the relatively small differences in mean workload) or at different sites (due to the large differences in ED workload rates across sites). In examining the ED workload rate, considerable variability also existed, which did not necessarily correlate with annual volume or number of ED beds available. To our knowledge, in the medical literature, no standardized measurement tool exists to compare EDs of differing capacity at the daily level. Thus, development of an ED workload rate fulfills a necessary role and shares many of the useful features of the occupancy rate, which has been validated elsewhere.

## LIMITATIONS

Sites were included in this study by a convenience sample of institutions where a local investigator was interested in ED crowding research and had access to electronic data. Although a community hospital was included, most sites were large, academic medical centers, which may reduce the generalizability of the findings to other settings. All of the sites were selected because they continue to use ambulance diversion. Sites that have abandoned diversion, including those in the state of Massachusetts, were not asked to participate in the study and would not find the results applicable to their practice environments.

Given the scope of this study, we were unable to include important factors like severity of disease, granular duration of diversion episodes, and staffing levels. In addition, we only collected data from sites at the daily level. As a result, we limited the primary analysis to variables where data were available from all sites. Of the 32 variables originally considered, 12 did not have data available from all participating sites. For instance, daily variations in staffing levels, which limit ED capacity, were not available from any site.

We also created a new measure, the ED workload rate, which has not been validated in other settings. Further analysis of this scale is warranted for validation in multiple settings along with predictors of diversion and LOS using these sites. Ideally, this workload rate would have used median LOS as part of its calculation, but given that only four of the nine sites were able to provide this variable, mean LOS was used instead. The use of median LOS has been recently recommended as a more accurate metric for LOS.^21^ It is likely that the skewed workload rate seen in Site 8 would be closer to the others if the median value had been used in the calculation.

Our primary outcome variable, the ED workload rate, did not incorporate boarding time, since not all sites were able to provide these data. Using LOS as part of the workload rate score indirectly accounts for delays on the output of admitted patients to inpatient locations. This is important, as the boarding of inpatients has been determined to be a primary factor driving ambulance diversion.^22,23^

Ambulance diversion was included as a dichotomous variable in the multivariate analysis of whether or not it occurred during a 24-hour period. As 1-hour of diversion is drastically different from 24 hours of diversion, this lack of granularity limits the conclusions that can be drawn from our analysis. However, a five-level diversion variable was used to further clarify the association between ED workload rate and diversion and demonstrated a logical dose–response relationship, speaking to the validity of ED workload rate as a standardized measure for workload and crowding. The distribution of daily hours on ambulance diversion (with extensive periods of no diversion) prohibited us from using daily hours of ambulance diversion simply as a continuous variable in the model. Finally, only association, not causation, can be inferred from the results.

## CONCLUSIONS

Given the variability among sites in the use of diversion, the question must be raised if ambulance diversion is a valid surrogate measurement to use when studying ED crowding. To date, ambulance diversion has been cited as being caused by crowding,^24^ but there might not be as much of a direct correlation as previously thought. This study demonstrated considerable variation in the use of ambulance diversion as a measure against the ED crowding burden when correlated to a daily workload rate. The observed variation may question the utility of ED diversion as an outcome variable for research studies that involve more than one ED with variable ED diversion criteria. Because some sites demonstrated significant differences in their ED workload rates and others did not, this suggests that ambulance diversion may not be generalizable as an outcome variable in ED crowding studies. Instead, measures like ED occupancy, median LOS, or elopements may be better markers of ED crowding.

## Figures and Tables

**Table 1:** Demographics of Sites

Site	Annual Census(2009)	Days With Ambulance Diversion (%)	ED Beds	Type of Practice	Mean Overall LOS (hours)
1	43,028	14.0	27–33	Academic	4.1
2	50,259	13.4	33	Community	5.0
3	56,756	43.8	48	Academic	5.2
4	57,911	86.6	45	Academic	7.6
5	59,453	5.5	48	Academic	4.7
6	62,186	75.1	48	Academic	7.2
7	85,066	4.9	56	Academic	4.9
8	98,214	71.0	95	Academic	21.6
9	101,236	16.7	44	Academic	5.3
All sites (mean)	68,234	36.8	50		7.3

**Table 2:** Workload Rates (Patient LOS Hours per ED Bed) for Days With and Without Ambulance Diversion by Sites

Site	Workload Rate on Days With Diversion			Workload Rate on Days Without Diversion			Adjusted Mean Difference in Workload Rate*	Percentage Increase in Workload Rates
	*N*	Crude	Adjusted	*N*	Crude	Adjusted		
1	51	17.1 (2.9)	15.9 (0.9)	314	14.7 (3.1)	14.3 (0.4)	1.6 (−1.0 to 4.2)	11.4
2	49	21.7 (2.8)	21.2 (0.9)	316	20.8 (2.9)	20.2 (0.4)	1.0 (−1.6 to 3.7)	5.1
3	160	18.8 (2.2)	17.4 (0.5)	205	15.3 (2.7)	15.3 (0.4)	2.2† (0.3 to 4.0)	14.2
4	316	27.5 (5.3)	26.7 (0.4)	49	22.3 (3.9)	22.8 (0.9)	3.9† (1.2 to 6.6)	17.1
5	20	20.4 (4.9)	19.2 (1.4)	345	15.8 (4.0)	15.2 (0.3)	4.0† (0.0 to 7.9)	26.1
6	274	26.8 (5.7)	25.9 (0.4)	91	22.5 (5.5)	22.6 (0.7)	3.3† (1.2 to 5.4)	14.5
7	18	25.2 (8.1)	23.1 (1.5)	347	20.3 (9.4)	19.8 (0.3)	3.3 (−0.9 to 7.5)	16.4
8	259	62.1 (11.8)	61.1 (0.4)	106	59.3 (11.5)	59.8 (0.6)	1.3 (−0.7 to 3.3)	2.2
9	61	39.6 (7.0)	38.0 (0.8)	304	32.4 (6.6)	32.0 (0.4)	6.0† (3.5 to 8.4)	18.7

Results are reported as mean (SE) or rate (95% CI).

* With Bonferroni correction.

† Statistically significant (p < 0.05).

**Table 3:** Subanalysis of Mean Boarding Time (Hours / Patient) for Days With and Without Ambulance Diversion by Selected Site

Site	Boarding Time on Days With Diversion			Boarding Time on Days Without Diversion			Adjusted Mean Difference in Boarding Time (Hours) (95% CI*)
	*N*	Crude	Adjusted	*N*	Crude	Adjusted	
1	51	1.3 (0.1)	1.0 (0.2)	314	1.1 (0.0)	0.8 (0.1)	0.2 (−0.3 to 0.6)
3	160	1.9 (0.1)	1.4 (0.1)	205	1.4 (0.0)	1.4 (0.1)	0.0 (−0.3 to 0.4)
5	20	1.8 (0.1)	3.4 (0.3)	345	3.9 (0.5)	1.6 (0.1)	1.7† (1.0 to 2.5)
6	274	4.7 (0.1)	4.4 (0.1)	91	2.9 (0.1)	2.9 (0.1)	1.5† (1.1 to 1.8)
9	61	5.5 (0.1)	4.9 (0.2)	304	4.2 (0.1)	4.0 (0.1)	0.9† (0.5 to 1.4)

Results are reported as mean (SE).

* With Bonferroni correction.

† Statistically significant (p < 0.05).
